# GC-MS-based untargeted metabolic profiling of malignant mesothelioma plasma

**DOI:** 10.7717/peerj.15302

**Published:** 2023-05-18

**Authors:** Ding Wang, Jing Zhu, Na Li, Hongyang Lu, Yun Gao, Lei Zhuang, Zhongjian Chen, Weimin Mao

**Affiliations:** 1 Zhejiang Cancer Hospital, Hangzhou Institute of Medicine (HIM), Chinese Academy of Sciences, Hangzhou, China; 2 The Second Clinical Medical College, Zhejiang Chinese Medical University, Hangzhou, China; 3 Key Laboratory Diagnosis and Treatment Technology on Thoracic Oncology, Hangzhou, China; 4 Shaoxing No. 2 Hospital Medical Community General Hospital, Shaoxing, China

**Keywords:** Malignant mesothelioma, Metabolomics, GC-MS, Biomarker

## Abstract

**Background:**

Malignant mesothelioma (MM) is a cancer caused mainly by asbestos exposure, and is aggressive and incurable. This study aimed to identify differential metabolites and metabolic pathways involved in the pathogenesis and diagnosis of malignant mesothelioma.

**Methods:**

By using gas chromatography-mass spectrometry (GC-MS), this study examined the plasma metabolic profile of human malignant mesothelioma. We performed univariate and multivariate analyses and pathway analyses to identify differential metabolites, enriched metabolism pathways, and potential metabolic targets. The area under the receiver-operating curve (AUC) criterion was used to identify possible plasma biomarkers.

**Results:**

Using samples from MM (*n* = 19) and healthy control (*n* = 22) participants, 20 metabolites were annotated. Seven metabolic pathways were disrupted, involving alanine, aspartate, and glutamate metabolism; glyoxylate and dicarboxylate metabolism; arginine and proline metabolism; butanoate and histidine metabolism; beta-alanine metabolism; and pentose phosphate metabolic pathway. The AUC was used to identify potential *plasma* biomarkers. Using a threshold of AUC = 0.9, five metabolites were identified, including xanthurenic acid, (s)-3,4-hydroxybutyric acid, D-arabinose, gluconic acid, and beta-d-glucopyranuronic acid.

**Conclusions:**

To the best of our knowledge, this is the first report of a plasma metabolomics analysis using GC-MS analyses of Asian MM patients. Our identification of these metabolic abnormalities is critical for identifying plasma biomarkers in patients with MM. However, additional research using a larger population is needed to validate our findings.

## Introduction

Malignant mesothelioma (MM) is a cancer associated with asbestos exposure, and is a rare and aggressive disease with a poor prognosis. Moreover, MM is an asbestos-related neoplasm that cannot be successfully treated unless detected and treated soon after its onset (Pass et al., 2020). Asbestos can drive the body to create induced inflammatory substances and damage genetic material; oxidative stress contributes to the creation of MPM (Pasello et al., 2018). The increase of malignant mesothelioma worldwide is approaching 94,000 cases every year (Strbac et al., 2017). Globally, the incidence of MM is expected to increase over the next decade because a long latency period exists between inhalation exposure and mesothelioma development (McCambridge et al., 2018; Halford & Clive, 2018; Shrestha et al., 2019). MM is usually diagnosed at advanced stages with a poor prognosis (Shrestha et al., 2019). After only supportive treatment, the median survival time is only 6–8 months, and after comprehensive treatment, the median survival time is only 12–16 months (McCambridge et al., 2018). Most patients will be required to undergo more invasive pleural procedures to detect mesothelioma, and imaging techniques often have difficulty detecting the disease (Halford & Clive, 2018). The efficacy of radiologic screenings is questionable and making a pathological diagnosis can be difficult (Viscardi et al., 2020). Therefore, the need for a novel and effective strategy to aid in diagnosis is urgent (McCambridge et al., 2018).

The development of biomarkers has become more important in the early detection of MM. Soluble mesothelin-related peptide is currently the only biomarker approved by the Food and Drug Administration to diagnose MM (Pass et al., 2020). Other biomarkers, such as osteopontin, fibulin-3, high mobility family box 1, microRNA, peripheral blood-based markers, and slow off-rate modified aptamers proteomics assays are expected to be used as biomarkers for early diagnosis of malignant mesothelioma. However, they all have their limitations, which involve standardization of measurement methods or the lack of accuracy due to the small number of samples (Sun, Vaynblat & Pass, 2017). This makes it necessary to identify additional novel and more efficient and accurate biomarkers.

Metabolomics, an emerging field founded on spectroscopic methods and technologies, is a valuable technique for conducting a complete and quantitative analysis of different metabolites in biological samples (Patel & Ahmed, 2015). With the in-depth use of metabolomics in the study of tumors, there have been significant breakthroughs in the identifications of biomarkers of many tumors such as pancreatic cancer, colorectal cancer, bladder cancer, *etc*. (Ni, Xie & Jia, 2014; Zhang et al., 2018; Long et al., 2018). At the same time, metabolomics also has its unique prospects in cancer research. Because metabolomics is very sensitive to small changes in biological pathways, it has the potential for discovering physiological conditions and abnormal processes (including underlying mechanisms of disease) (Johnson, Ivanisevic & Siuzdak, 2016). The metabolic profile of cancer cells shows the molecular biology of malignant mesothelioma, which helps find potential therapeutic targets.

In the present study, we aimed to identify novel metabolic markers that could improve the early diagnosis of MM. Gas chromatography-mass spectrometry (MS)-based untargeted metabolomics analyses were used to comprehensively screen plasma samples containing altered metabolites from both MM and healthy control (HC) groups. Receiver operating characteristic (ROC) curves were then analyzed using univariant analyses to detect biomarkers with high diagnostic capabilities for MM.

## Materials and Methods

### Study design and population

**Figure 1:**
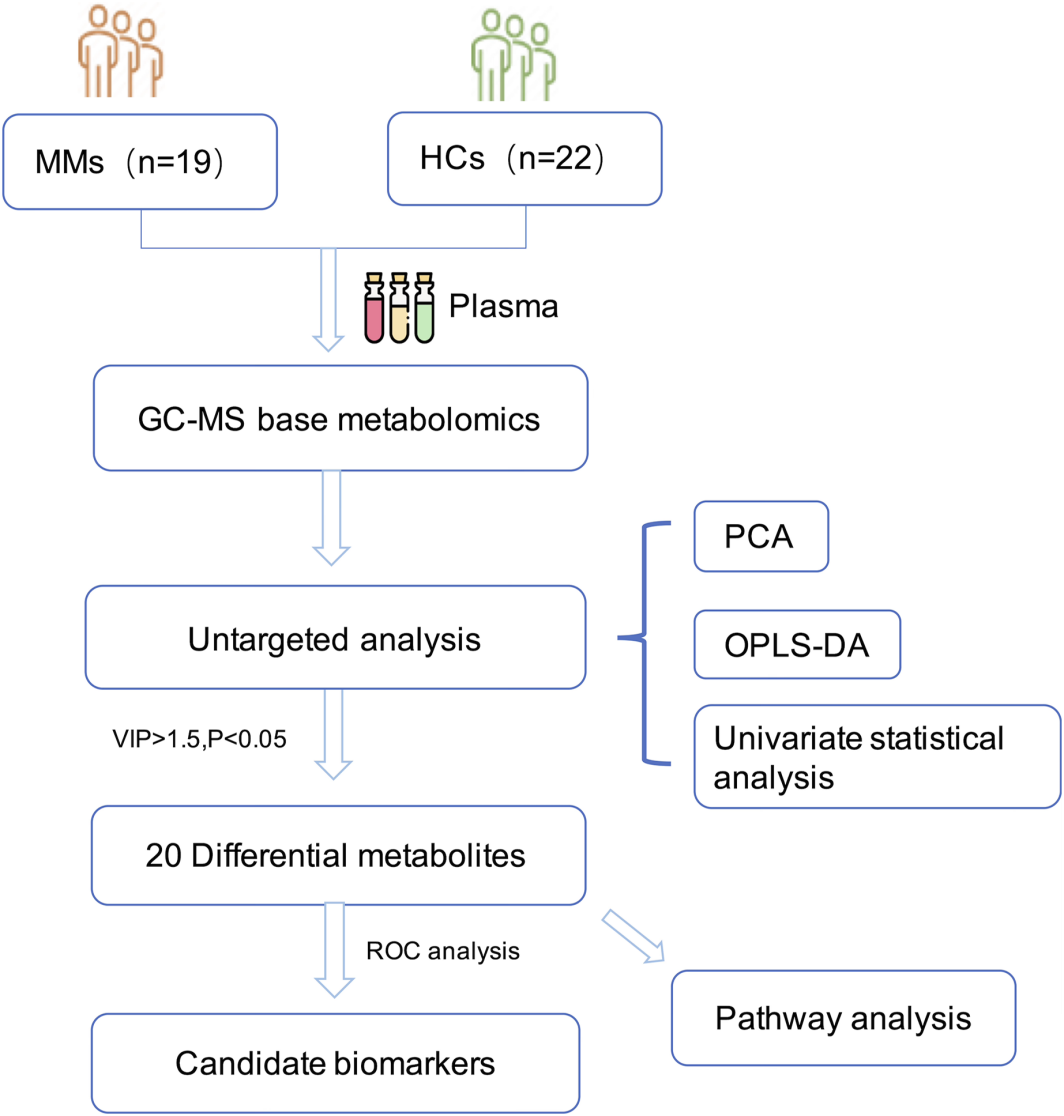
Diagram of the study.

The research was undertaken in compliance with the 1964 Declaration of Helsinki and was authorized by the Institutional Review Board and the ethical requirements of the Ethical Committee of Zhejiang Cancer Hospital. In accordance with the informed consent exemption rules of the Ethical Committee of Zhejiang Cancer Hospital (IRB-2021-65), our study complies with the informed consent exemption requirement. The plasma samples were taken from 19 patients with histopathologically confirmed MM at Zhejiang Cancer Hospital in China between March 2016 and March 2020. None of the patients had previously received any anti-cancer therapy. Control samples were taken from 22 age-matched and sex-matched HCs. All participants fasted overnight before sample collection. The plasma samples were then immediately centrifuged at 3,000 g for 15 min at 4 °C. Potassium diethylenediaminetetraacetic acid was used as an anticoagulant. Plasma samples were stored at −80 °C until analysis. The workflow of the study is described in (Fig. 1).

### Chemicals

Chemicals and solvents of analytical or HPLC grade were used. Thermo Fisher Scientific (Waltham, MA, USA) provided the acetonitrile and methanol. CNW Technologies (Düsseldorf, Germany) provided N,O-Bis(trimethylsilyl)trifluoroacetamide (BSTFA) with 1% trimethylchlorosilane (TMCS), n-hexane, methoxylamine hydrochloride (97%), and pyridine. Shanghai Heng Chuang Biotechnology (Shanghai, China) provided the L-2-chlorophenyl alanine.

### Sample preparation

The samples were stored at a temperature of −80 °C. After being thawed at room temperature, 80 μL of the sample was added to a 1.5 mL Eppendorf tube with 1–2 μL of L-2-chlorophenylalanine (0.3 mg/mL) dissolved in methanol as internal standard, and the tube was vortexed for 10 s. Next, 240 μL of acetonitrile (2/1, vol/vol) and ice-cold mixture of methanol were added. After vortexing for 1 min, the mixtures were extracted by ultrasonic for 10 min in an ice-water bath and then stored at −20 °C for 30 min. The extract was centrifuged at a temperature of 4 °C (13,000 rpm) for 10 min. A total of 150 uL of the supernatant in a glass vial was dried using a freeze concentration centrifugal dryer. In a centrifugal dryer with freeze concentration, all samples were evaporated. Subsequently, 80 uL of 15 mg/mL methoxylamine hydrochloride in pyridine were added. After vortexing for 2 min, the mixture was incubated at 37 °C for 90 min. Then, 20 μL n-hexane and 50 μL of BSTFA (with 1% TMCS) were added, the mixture vortexed vigorously for 2 min and then derivatized at 70 °C for 60 min. Before GC-MS analysis, samples were kept at ambient temperature for 30 min.

Additionally, pooled quality control samples (QC) were prepared by pooling aliquots from each MM and HC sample, then extracting them as described above. Equipment and steps used in the GC-MS analysis process can be seen in the supplementary information.

### Data preprocessing and statistical analysis

The GC/MS data were initially in an unprocessed form. The raw GC/MS data was converted using the Analysis Base File Converter software (https://www.reifycs.com/AbfConverter/). The data were then entered into the MS-DIAL software for preprocessing. Metabolites were characterized using LUG database (Lumingbio Untargeted database of GC-MS) (He et al., 2022; Miao et al., 2022), and a data matrix was derived. The three-dimensional matrix includes: sample information, the peak name for each substance, retention time, retention index, mass-to-charge ratio, and signal intensity. In each sample, all peak signal intensities were segmented and normalized according to the internal standards with a relative standard deviation (RSD) greater than 0.3 after screening.

The matrix was imported into R (The R Foundation for Statistical Computing, https://www.r-project.org/) so that principle component analysis (PCA) could be used to determine the overall distribution of samples and the general stability of the analysis. Orthogonal partial least squares-discriminant analysis (OPLS-DA) and partial least squares-discriminant analysis (PLS-DA) were used to differentiate the metabolites that differed between groups. A 7-fold cross-validation and 200 Response Permutation Testing were used to assess the model’s quality.

The variable importance of projection (VIP) values were used to rank the overall contribution of each variable to the group discrimination. A two-tailed Student’s t-test was used to determine the significance of the differences between groups. Different metabolites were selected that had VIP values > 1.5 and *p*-values < 0.05.

Biomarker analysis was conducted by using a classical univariate ROC curve, which was created by plotting the curves for true positive rate and false positive rate under different thresholds. Normally, the area under the ROC curve (AUC) is used as a method to assess the overall diagnostic accuracy of a particular index in disease diagnosis. The standard of AUC value is: 0.5 < AUC < 0.7, the diagnostic accuracy is low, 0.7 < AUC < 0.9 represented a moderate diagnostic accuracy, and the diagnostic accuracy of AUC > 0.9 is high.

### Pathway analysis

Metabolic pathway analysis was performed using the MetPA tool in MetaboAnalyst (version 5.0, https://www.metaboanalyst.ca/) to understand the biological significance of our findings from both the MM and HC groups. MetPA is a pathway analysis tool that combines two approaches involving enrichment and topology analysis. The Human Metabolome Database and the Kyoto Encyclopedia of Genes and Genomes databases were both used to identify genes associated with differentially-expressed metabolites. For each pathway, a *p*-value and an impact score were calculated.

## Results

### Clinical characteristics of the participants

The analysis included a total of 41 participants divided into two groups involving MM (*n* = 19) and HCs (*n* = 22). In terms of age and sex, there was no statistically significant intergroup difference (*p*-value = 1.00), as shown in (Table 1). There were 13 pleural MM patients and six peritoneal MM patients. In terms of asbestos exposure, seven patients were positive and 12 were negative.

**Table 1:** The clinical features of patients with malignant mesothelioma (MM) and healthy control (HC).

Feature	HC (*n* = 22)	MM (*n* = 19)	*p*-value
Age			
Mean ± SD	57.64 ± 8.13	57.84 ± 9.29	0.94
Gender			
Male	12 (54.5%)	11 (57.9%)	1.00
Female	10 (45.5%)	8 (42.1%)	
Site	NA		
Pleural mesothelioma		13 (68.4%)	
Peritoneal mesothelioma		6 (31.6%)	
Asbestos exposure	NA		
Yes		7 (36.8%)	
No		12 (63.2%)	

**Note:**

HC, healthy control group; MM, malignant mesothelioma group; Two tailed Chi-square test was used to compare the distribution of sex or age between two groups, *p*-value < 0.05 was recognized as significant. NA, not available.

### Untargeted metabolomics profiles of plasma between the MM and HC groups

**Figure 2:**
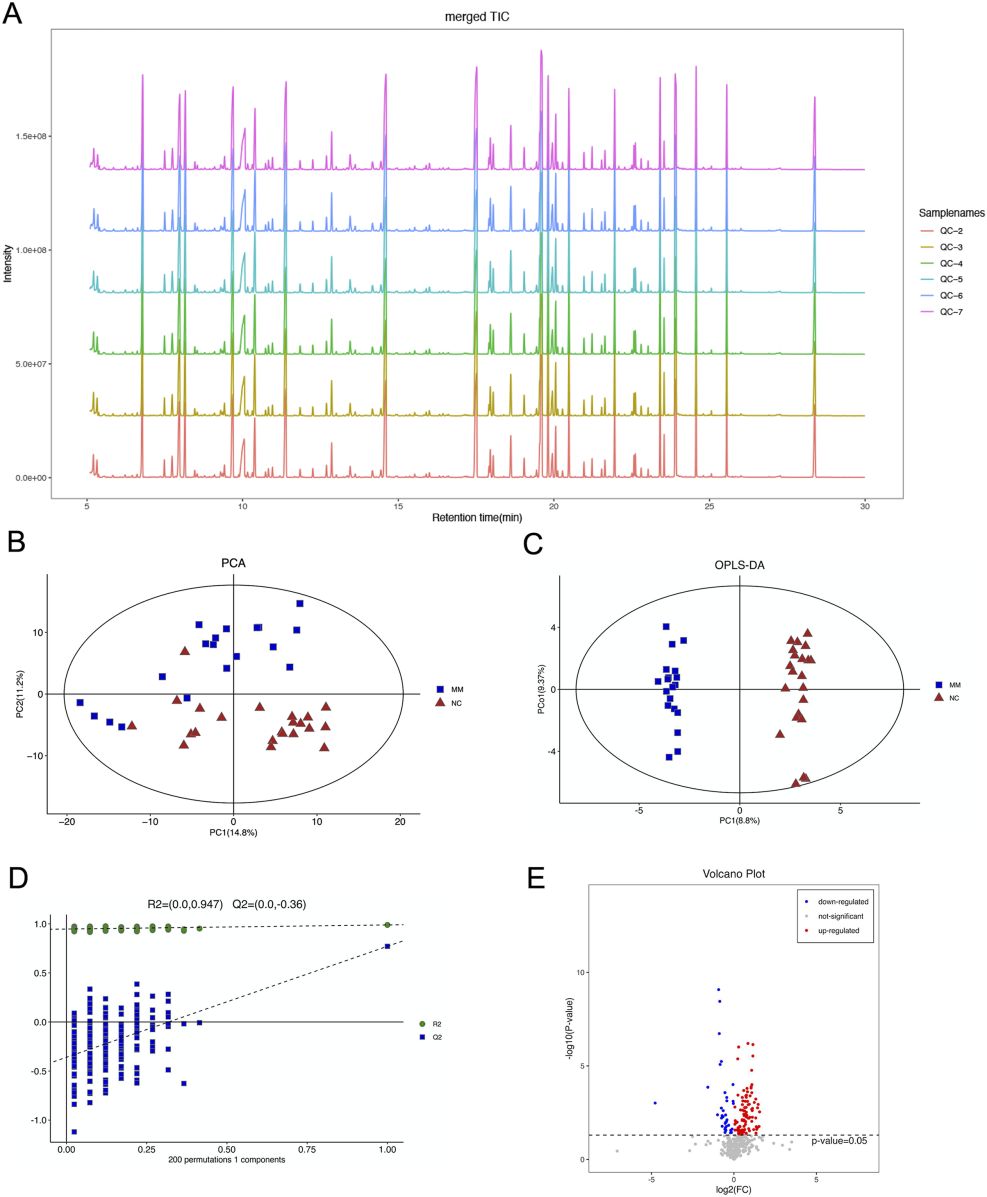
Gas chromatography-mass spectrometry and metabolomics analyses were used to identify metabolic features that differed between the malignant mesothelioma (MM) and healthy control (HC) groups. (A) The total ion flow diagrams of quality control samples were compared. (B) The plot of principle component analysis scores for all samples. (C) The plot of the orthogonal partial least squares-discriminant analysis scores for the MM and HC groups. (D) Permutation test performed after 200 repetitions. (E) Volcano plot for dysregulated ions using *p* < 0.05.

The total ion flow diagram of QC samples is compared by spectral overlap (Fig. 2A). Overall, the results showed that the response intensities and retention times of the peaks essentially overlapped, indicating that instrumental error had an insignificant impact during the experiments.

PCA is an unsupervised data analysis method that transforms the original random vectors, with components correlated into new random vectors whose components are not correlated with each other with the help of an orthogonal transformation. They reflect as much information of the original variables as possible, and thus achieve dimensionality reduction. The stability of the system was evaluated, as shown in Fig. 2B.

OPLS-DA is a supervised statistical procedure for performing discriminant analysis involving orthogonal partial least squares. There are two kinds of principal components using OPLS-DA score plots (Fig. 2C), which are predictive and orthogonal. The predictive principal component has only one component, while the orthogonal principal component can have multiple components. OPLS-DA maximizes the between-group variations reflected on t1. Therefore, between-group variations can be clearly distinguished from t1, while within-group variations can be discerned from the orthogonal principal component. The OPLS-DA score plots showed significant differences between the two groups.

The OPLS-DA model had R^2^X, R^2^Y and Q2 of 0.306, 0.989 and 0.771, respectively. The OPLS-DA analysis of train set samples showed a significant difference between the MM and HC groups, which was cross-validated with 200-time permutation tests (Fig. 2D). MM plasma samples contained a panel of dysregulated metabolic characteristics, with 95 upregulated and 37 downregulated parameters, according to a volcano map based on *p*-values (Fig. 2E).

### Differential metabolite screening

To identify metabolites that differed between groups, we conducted multidimensional and unidimensional analyses. VIP during OPLS-DA analysis was used to measure the intensity and explanatory power of the expression pattern of each metabolite on the categorical discrimination of each group of samples, to identify biologically significant differential metabolites. Furthermore, the *t*-test was used to ascertain whether there was a significant difference of the metabolites between groups.

The screening criteria were a VIP value > 1.5 for the first principal component of the OPLS-DA model and a *p*-value < 0.05 for the *t*-test. There were 20 metabolites that were annotated, with 15 of them being upregulated and five being downregulated (Table 2).

**Table 2:** Twenty distinct circulating metabolites between malignant mesothelioma patients and healthy controls were annotated.

No.	Metabolite	VIP^a^	*p*-value^b^	FC^c^	AUC^d^
1	Butane-2,3-diol	4.03	9.67E−04	0.04	0.86
2	3-hydroxypropionic acid	3.51	1.88E−03	2.18	0.83
3	(s)-3,4-dihydroxybutyric acid	2.27	7.24E−07	2.22	0.95
4	Gluconic acid	2.26	1.75E−03	2.7	0.92
5	Xanthurenic acid	2.24	8.29E−10	0.53	0.96
6	Beta-d-glucopyranuronic acid	2.04	1.01E−04	2.13	0.92
7	Taurine	1.98	1.15E−03	2.78	0.78
8	Aconitic acid	1.94	6.33E−07	1.8	0.89
9	4-aminobutyric acid	1.93	1.52E−04	2.06	0.88
10	L-histidine	1.91	6.18E−03	0.6	0.79
11	Glycocyamine	1.86	5.77E−06	0.59	0.86
12	Allantoic acid	1.84	1.72E−02	2.87	0.77
13	Azelaic acid	1.82	2.70E−04	2.12	0.83
14	2-monoolein	1.76	1.30E−04	2.07	0.82
15	N-carbamoylaspartate	1.74	4.13E−03	0.5	0.77
16	L-2-hydroxyglutaric acid	1.72	3.92E−04	1.65	0.86
17	Glyceric acid	1.64	1.64E−03	1.67	0.83
18	D-arabinose	1.62	4.43E−03	1.72	0.92
19	Glucose	1.6	3.98E−04	1.97	0.74
20	L-glutamic acid	1.52	5.39E−03	1.68	0.74

**Notes:**

a Variable importance in the projection of OPLS-DA model.

b From two-tailed Student’s t-test test.

c Fold change (MM *vs* control).

d The area under ROC curve.

**Figure 3:**
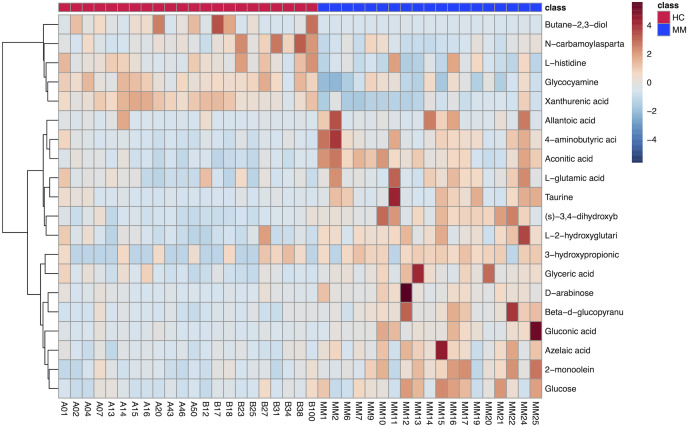
Heat map of 20 differentially expressed metabolites between the malignant mesothelioma and healthy control groups.

The results of hierarchical clustering are shown in Fig. 3, which shows the relationship between samples and the variations of levels of metabolites between samples. The horizontal coordinates indicate the sample names, and the vertical coordinates indicate the differential metabolites. The color of the expression abundance of metabolites from blue to red indicates the level of expression abundance, *i.e*., the darker red indicates better expression abundance of differentially expressed metabolites.

**Figure 4:**
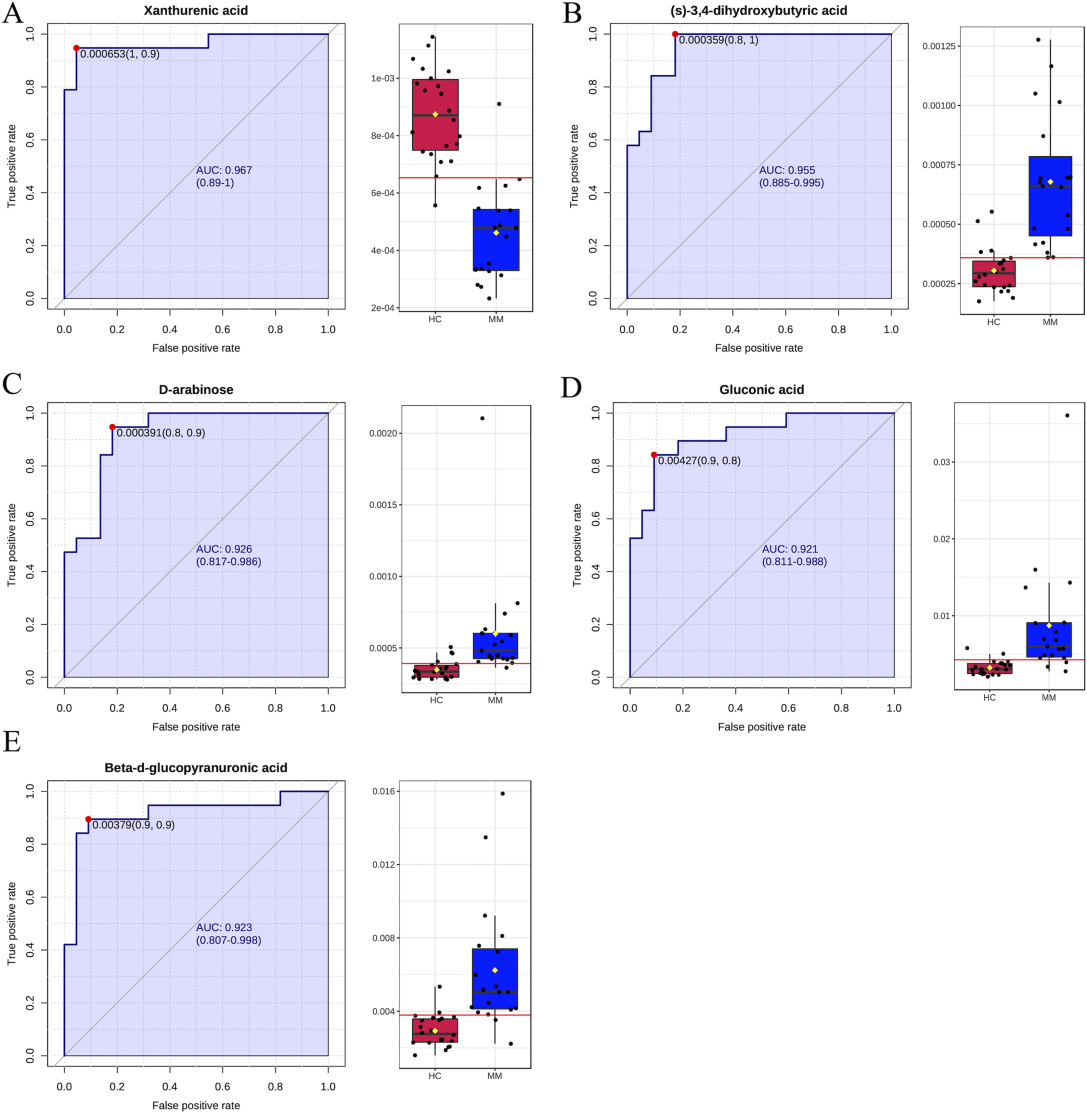
Receiver operating characteristic (ROC) curves for five differential metabolite data sets with the area under the curve >0.9 based on peak intensities or prediction probabilities. (A–E) ROC curve analyses of the ability of five metabolites to predict malignant mesothelioma (MM) *vs* healthy controls (HCs).

Finally, ROC curve analysis was used to assess the diagnostic ability of differentiated metabolites from each volcano plot as MM screening biomarkers. The x- and y-axes represent 1-specificity and sensitivity, respectively. The results indicated that the AUCs for five metabolites were greater than 0.9 in the MM group when compared with the HC group (Fig. 4), including xanthurenic acid (AUC: 0.969), (s)-3,4-dihydroxybutyric acid (AUC: 0.957), D-arabinose(AUC: 0.922), gluconic acid (AUC: 0.921), beta-d-glucopyranuronic acid (AUC: 0.923).

### Pathway analysis

**Figure 5:**
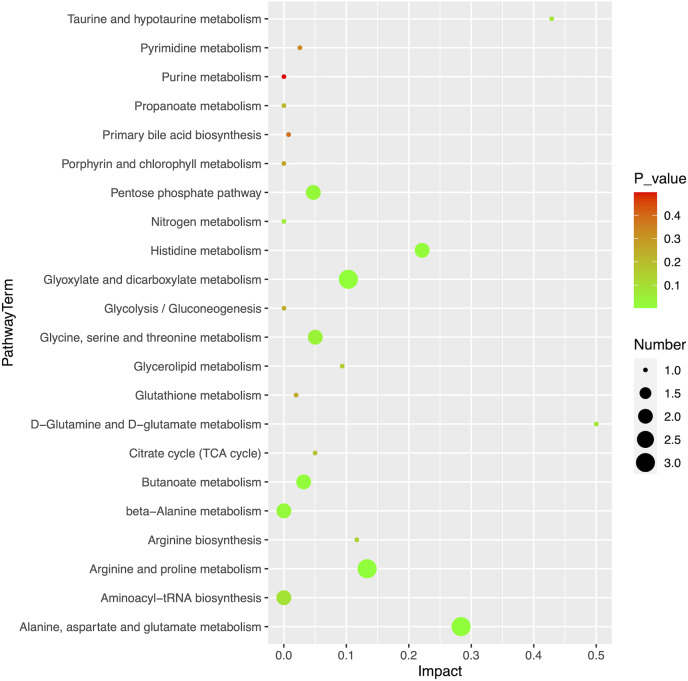
Pathway analysis of 20 differential metabolites.

Using MetaboAnalyst (version 5.0, https://www.metaboanalyst.ca/), we obtained an overview of the pathway impacts of attributed metabolites (MM *vs* HC) (Fig. 5). Furthermore, 22 pathways were enriched for these metabolites, the majority of which were related to amino acid metabolism, with seven pathways exhibiting statistically significant differences with values of *p* < 0.05, including alanine, aspartate, and glutamate metabolism (*p* = 0.0104); glyoxylate and dicarboxylate metabolism (*p* = 0.0151); arginine and proline metabolism (*p* = 0.0240); butanoate metabolism (*p* = 0.0249); histidine metabolism (*p* = 0.0281); beta-alanine metabolism (*p* = 0.0467); and pentose phosphate pathway (*p* = 0.0257) (Table 3).

**Table 3:** Plasma differential metabolite pathway analysis between the malignant mesothelioma and healthy control groups.

Pathway name	Total^a^	Hits^b^	*p*-value	−log10^P^	Rich factor^c^
Alanine, aspartate and glutamate metabolism	28	3	2.53E−03	2.597	0.284
Glyoxylate and dicarboxylate metabolism	32	3	3.73E−03	2.428	0.103
Arginine and proline metabolism	38	3	6.11E−03	2.214	0.133
Butanoate metabolism	15	2	9.70E−03	2.013	0.032
Histidine metabolism	16	2	1.10E−02	1.958	0.221
beta-Alanine metabolism	21	2	1.87E−02	1.728	0.000
Pentose phosphate pathway	22	2	2.05E−02	1.689	0.047

**Notes:**

a Number of total metabolites in pathway.

b Number of metabolites detected.

c Ratio of detected metabolites in the pathway. Result from online tool (https://www.metaboanalyst.ca).

## Discussion

MM primarily originates from the pleura, followed by the peritoneum, the pericardium, and the reproductive system. Malignant pleural mesothelioma (MPM) is rare and occurs mostly in men, accounting for 2% and 5% of all pleural malignant tumors. The diagnosis of mesothelioma has traditionally been considered a challenging task, although immunohistochemical staining has made it more likely to be accurate (Husain et al., 2013). The majority of patients are diagnosed with advanced stages of the disease and have a median survival time of less than 12 months (Zucali et al., 2011), but an early diagnosis of MM has been shown to improve overall survival (Pass et al., 2020). With the increasing incidence of MPM and its long latency periods, earlier diagnosis and better prognosis are therefore imperative.

We used GC-MS of untargeted metabolomics to identify differential metabolites between plasma samples of MM patients and age- and sex-matched HCs, aiming to enhance MM prognosis by optimizing the clinical diagnosis. A total of 20 metabolites were identified as dysregulated after screening and annotation. We also used ROC models based on a single metabolite to test how important these metabolites were for diagnosis.

Proliferation of cancer cells requires an abundant supply of amino acids (Vettore, Westbrook & Tennant, 2020). Along with their direct role in protein synthesis, nucleosides are also essential for energy generation, nucleoside synthesis, and redox homeostasis in cells. It is not unusual for cancer cells to be part of a metabolic process in nutrient-poor microenvironments, forming relationships that can be both symbiotic and parasitic. This is particularly evident in cancers that are auxotrophic for certain amino acids. After pathway analyses of the different metabolites, we focused on amino acid metabolism. According to Chen et al. (2015), there were differences between the metabolic profiles of colorectal polyp patients and controls. Using the seemingly unrelated regression created from the NMR data of sera, they found that polyps had abnormal alanine, aspartate, and glutamate metabolisms. In the present study, we also found abnormal metabolic changes in alanine, aspartate, and glutamate metabolisms by using GC-MS non-targeted metabolites. Wang et al. (2021) also reported abnormal metabolic changes in alanine, aspartic acid, and glutamate in epithelial ovarian cancers. We speculate that the metabolic pathways involving alanine, aspartate, and glutamate may have a role in the occurrence and progression of mesothelioma.

Glycolysis is a central step of carbohydrate metabolism, and plays a vital role in cancer development and aging (Parkhitko et al., 2020). A distinct feature of tumor cells involves the change in carbohydrate metabolism to aerobic glycolysis. It is well known that even in the presence of oxygen, cancer cells utilize glucose significantly more rapidly than they would otherwise, which is known as the “Warburg effect”. The Warburg effect is an essential metabolic characteristic of many types of cancer. BRCA1-related protein 1 (BAP1) plays a vital role in regulating environmental carcinogenesis. A mutation in BAP1 is a frequent somatic event in peritoneal MM (Alakus et al., 2015). A number of biological processes are regulated by BAP1, including chromatin modification, cell cycle, apoptosis, ferroptosis, cell metabolism, and differentiation (Carbone et al., 2020). Moreover, BAP1^+^ fibroblasts enhance aerobic glycolysis and lactate secretion, but reduce mitochondrial respiration and ATP production, when compared with wild-type BAP1. In the present study, there were two pathways involving significant changes related to carbohydrate metabolism after pathway analysis, including glyoxylate and dicarboxylate metabolism and butanoate metabolism. The metabolic changes related to carbohydrate metabolism included 4-aminobutyric acid, L-glutamic acid, aconitic acid, and glyceric acid. Compared to the HC group, the level of glyceric acid significantly increased in the MM group. As an intermediate in serine degradation, glyceric acid is phosphorylated to produce glycerate 3-phosphate, a component of glycolysis, which provides energy to tumor cells. A previous study reported that patients with advanced pancreatic cancer experienced a decrease in glyceric acid levels in their blood (Nishiumi et al., 2010). Furthermore, Ikeda et al. (2012) confirmed that patients with esophageal cancer, gastric cancer, and colorectal cancer had lower levels of lever glyceric acid in their plasma, when compared with controls. However, unlike other tumors, glyceric acid levels are high in patients with MM, which may be because it plays a part in the enhancement of tumor metabolism, so it shows some stability as a diagnostic marker. Thus, targeting glycolysis pathway may be a new research direction in the treatment of patients with malignant mesothelioma.

We also found abnormal changes of histidine metabolism in the MM group. Histidine is a component of protein-based dairy and meat products. As Rudolph Virchow first observed, tumors arise at sites of chronic inflammation due to the presence of inflammatory cells (Schetter, Heegaard & Harris, 2010). The study of Feng et al. (2013) has shown that histidine supplementation can inhibit inflammation and oxidative stress. Liu et al. (2015) reported that calcium-binding proteins with histidine promoted the growth of hepatocellular carcinomas, and the MEK/ERK pathway was essential to HRC-induced enhancement of cell proliferation. Li et al. (2021) reported that the relative abundance of the L-histidine metabolic pathway was related to thyrotropin-releasing hormone in patients with high grade thyroid nodules. Froldi et al. (2019) reported that changes in histidine metabolism exclusively targeted the formation of neural tumors. Ni, Xie & Jia (2014) observed that histidine levels were lower in patients with colorectal cancer than in normal controls. In addition, studies by Kanarek et al. (2018) have suggested that the histidine degradation pathway significantly affects the sensitivity of cancer cells to methotrexate, which can be improved by simple dietary intervention. The plasma levels of histidine in patients with MM were significantly lower, and metabolic pathways were abnormal. Accordingly, we speculate that abnormal histidine metabolism may be related to the occurrence and development of MM and that histidine supplementation may benefit patients with mesothelioma.

This study had some limitations. The sample size was quite modest in comparison to other studies using GC-MS-based learning methods. As a result of the small sample size, this study may only be considered a pilot study. A larger cohort is required to validate this unique method of diagnosing MM. Furthermore, due to the rarity of MM, high sample size-based metabolomics may require collaboration between different centers. In our study, we selected VIP > 1.5 and *p* < 0.05 as the criteria for identifying differential metabolites, which may have some impact on the results. By using a VIP > 1.5 threshold, our findings may exhibit greater significance compared to employing a VIP > 1 screening standard. However, this approach might also result in a more stringent selection criterion for differential metabolites, potentially causing the exclusion of some valuable research outcomes. Furthermore, we noticed that some of our hypothesis tests were performed without adjusting for multiple comparisons, which could lead to false positive results. Moving forward, we will investigate more appropriate criteria for determining differential metabolites and enhance the correction of hypothesis test results.

In conclusion, MM is an aggressive and rare malignancy associated with chronic inflammation and oxidative stress-induced predominantly by asbestos exposure. The plasma metabolic profiles of MM patients were studied for the first time using GC-MS, to find multiple dysregulated metabolism pathways. Among the dysregulated pathways were alanine, aspartate, and glutamate metabolism; glyoxylate and glyoxylate metabolism; arginine and proline metabolism; butanoate metabolism; histidine metabolism; and beta-alanine metabolism, which provided insight into the molecular biology of MM, as well as providing potential therapeutic targets for MM. Furthermore, the diagnostic value for MM revealed a novel and successful technique for MM diagnosis. It prompted a further study with a higher sample size, which could result in a favorable impact on the prognoses of MM patients.

## Supplemental Information

10.7717/peerj.15302/supp-1 Supplemental Information 1 GC-MS data. Plasma metabonomic analysis between patients with malignant mesothelioma and healthy controls was based on raw data above.

10.7717/peerj.15302/supp-2 Supplemental Information 2 Detailed information on chemicals, equipment, and chromatography and mass spectrometry analytical conditions used in the GC-MS analysis.

## Additional Information and Declarations

### Data Availability

The following information was supplied regarding data availability: The data are available in the Supplemental Files.
