# Supplementary material for: GC-MS-based untargeted metabolic profiling of malignant mesothelioma plasma
Source: PeerJ. 2023 May 18;11:e15302. doi: 10.7717/peerj.15302 (PMC10200095; doi:10.7717/peerj.15302)
Supplement: Supplemental Information 2 [file peerj-11-15302-s002.docx]

**Supplemental information**

**Chemical regents**

Chemicals and solvents of analytical or HPLC grade were used. Thermo Fisher Scientific (Waltham, MA, USA) provided the acetonitrile and methanol. CNW Technologies (Düsseldorf, Germany) provided N,O-Bis(trimethylsilyl)trifluoroacetamide (BSTFA) with 1% trimethylchlorosilane (TMCS), n-hexane, methoxylamine hydrochloride (97%), and pyridine. Shanghai Heng Chuang Biotechnology (Shanghai, China) provided the L-2-chlorophenyl alanine

**Use of other equipment**

Ultrasonic Cleaner (SB-5200DT, Ningbo Xinzhi Biological Technology Co., Ltd.), vortex oscillator (TYXH-I, Shanghai Hannuo Instrument Co., Ltd.), Freeze-concentrated centrifugal dryer(LNG-T98, Taicang Huamei Biochemical Instrument Factory), High-speed refrigerated centrifuge (TGL-16MS, Shanghai Lu Xiangyi Instrument Co., Ltd.), Gas bath constant temperature oscillator (THZ-82A, Jiangsu Huanyu Scientific Instrument Factory), Vacuum drying oven (DZF-6021,Shanghai Huitai Co., Ltd.).

**Chromatographic conditions are as follows:**

DB-5MS capillary column (30 m × 0.25 mm × 0.25 μm, Agilent J&W Scientific, Folsom, CA, USA) was used as the stationary phase. High purity helium gas (purity not less than 99.999%) was used as the carrier gas with a flow rate of 1.0 mL/min. The temperature of the inlet was set at 260°C. The injection volume was 1μL without splitting, and the solvent delay was 5 min.

The temperature program was as follows: the initial temperature of the column oven was set at 60°C and held for 0.5 min. Then the temperature was increased at a rate of 8°C/min to 125°C, followed by an increase at a rate of 8°C/min to 210°C. Next, the temperature was increased at a rate of 15°C/min to 270°C and finally increased at a rate of 20°C/min to 305°C and held for 5 min.

**Mass spectrometric conditions are as follows:**

Electron ionization source (EI) was used with the ion source temperature set at 230°C and the quadrupole temperature set at 150°C. The electron energy was set at 70 eV. The scan mode was set to full scan mode (SCAN), and the mass scanning range was m/z 50-500.
